# Supplementary material for: Tumor-Infiltrating B Cells and Tissue-Resident Memory T Cells as Prognostic Indicators in Brain Metastases Derived from Gastrointestinal Cancers
Source: Cancers (Basel). 2024 Nov 8;16(22):3765. doi: 10.3390/cancers16223765 (PMC11591993; doi:10.3390/cancers16223765)
Supplement: Supplementary file 1 [file cancers-16-03765-s001.zip › cancers-3149107-supplementary.pdf]

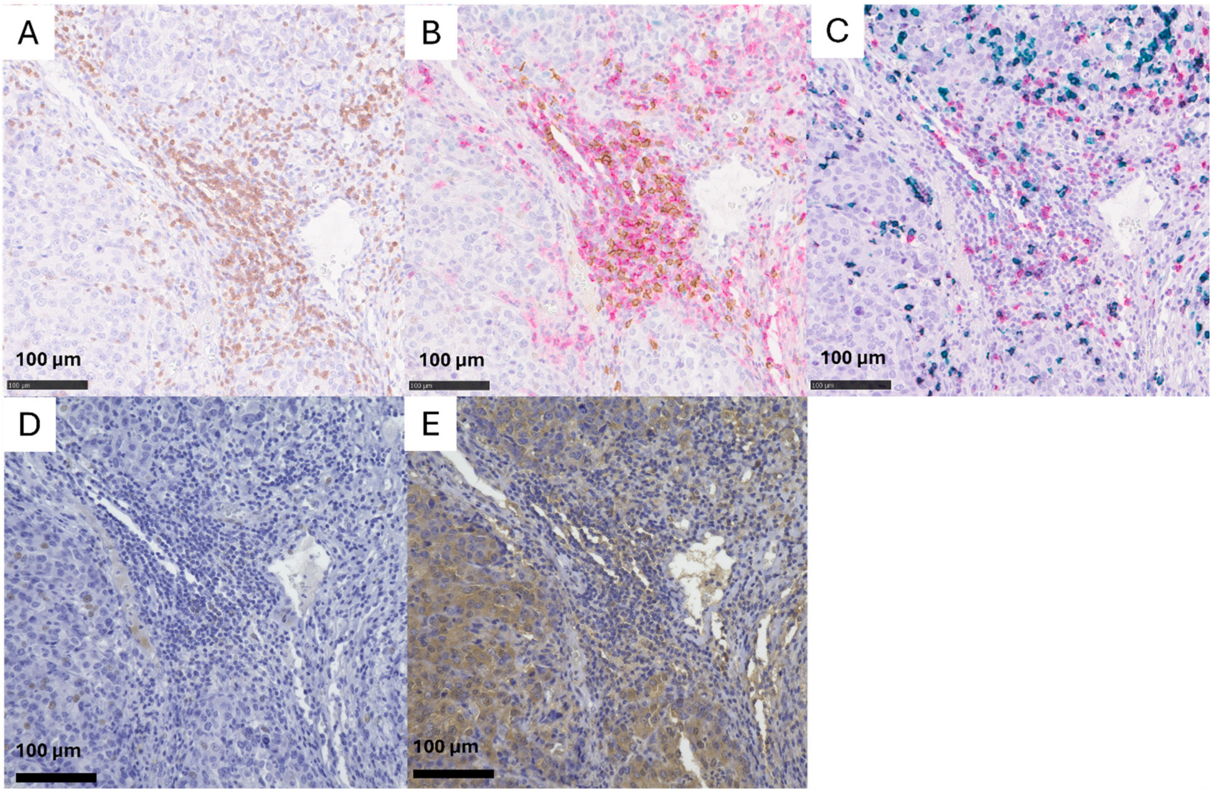

Figure S1. Immunohistopathological staining of lymphocyte subsets and key components of TLSs in BMs derived from gastrointestinal cancers.

Representative immunohistochemistry images of consecutive tissue sections from metastatic brain tumors. A: CD3 was stained using DAB (brown). B: CD20 (brown), CD4 (red), and BCL6 (green) were simultaneously stained. C: CD103 (green) and CD8 (red) were simultaneously stained. D: CD21 was stained using DAB (brown). E: PNAd was stained using DAB (brown). Despite the presence of CD20+ B cell and CD4+ T cell clusters (B), the lymphocyte aggregates in BMs lack CD21+ follicular dendritic cells (D) and PNAd+ high endothelial venules (E), which are key components of TLSs. Scale bars = 100 µm.

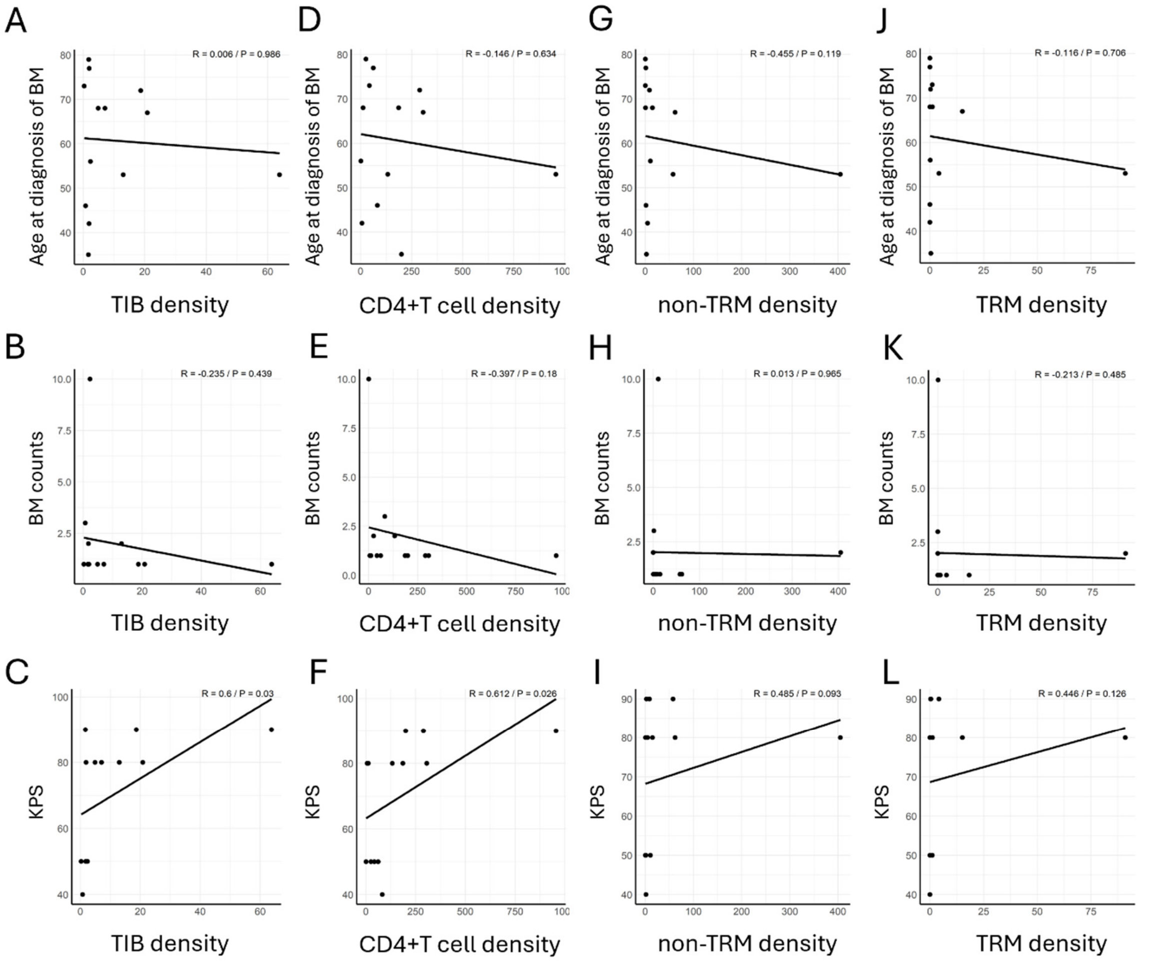

Figure S2. Correlations between densities of each TIL subset and clinical parameters.

Spearman's rank correlation coefficient analyses between densities of TIBs (A-C), CD4+ T cells (D-F), non-TRMs (G-I), or TRMs (J-L) and age at diagnosis of BM (A, D, G, J), BM counts (B, E, H, K), and KPS (C, F, I, L).

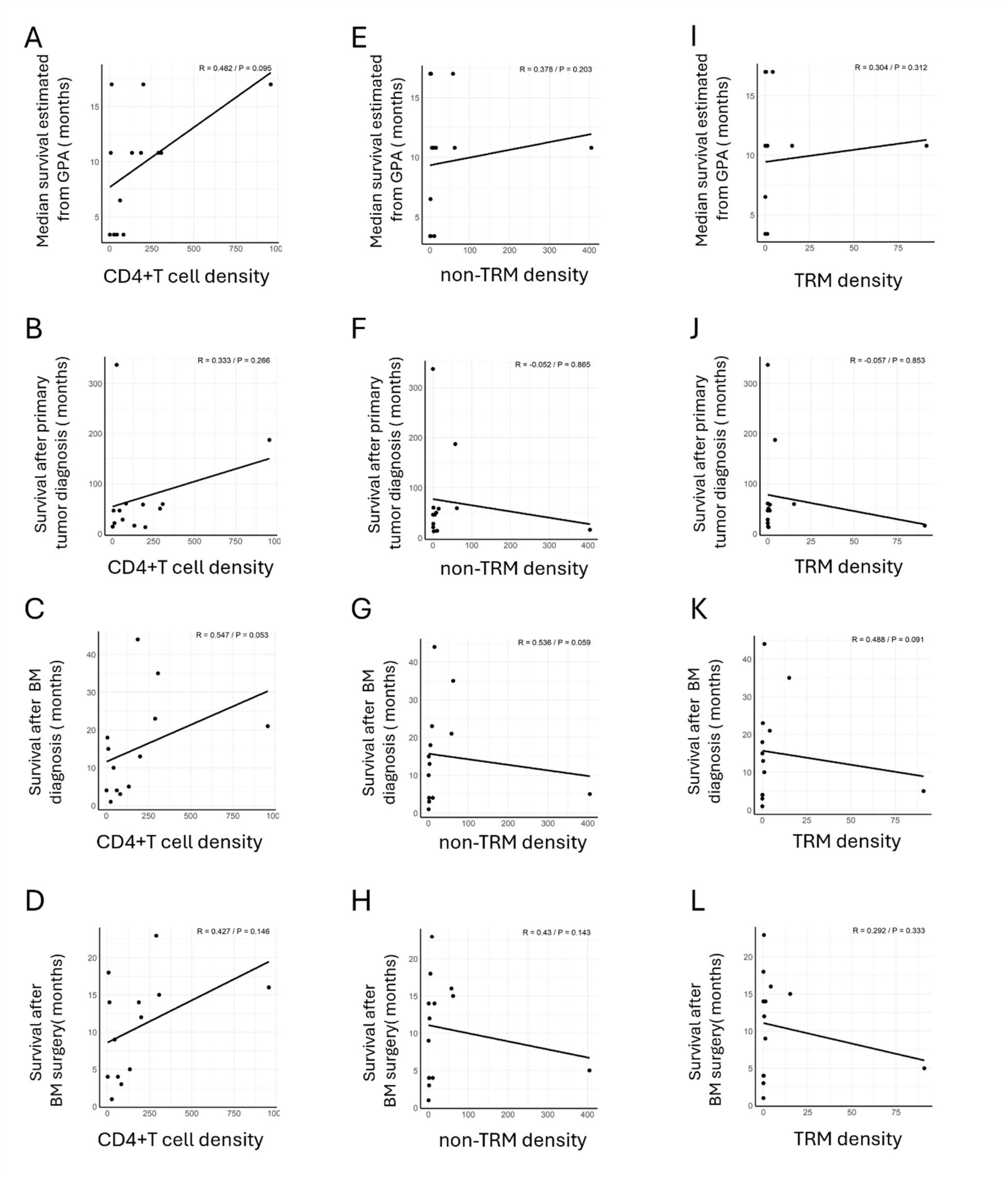

Figure S3. Correlations between densities of each TIL subset and OS including GPA-estimated survival.

Spearman's rank correlation coefficient analyses between densities of CD4+ T cells (A-D), non-TRMs (E-H), or TRMs (I-L) and OS after the estimated survival by GPA (A, E, I), primary lesion diagnosis (B, F, J), BM diagnosis (C, G, K), and BM surgery (D, H, L).

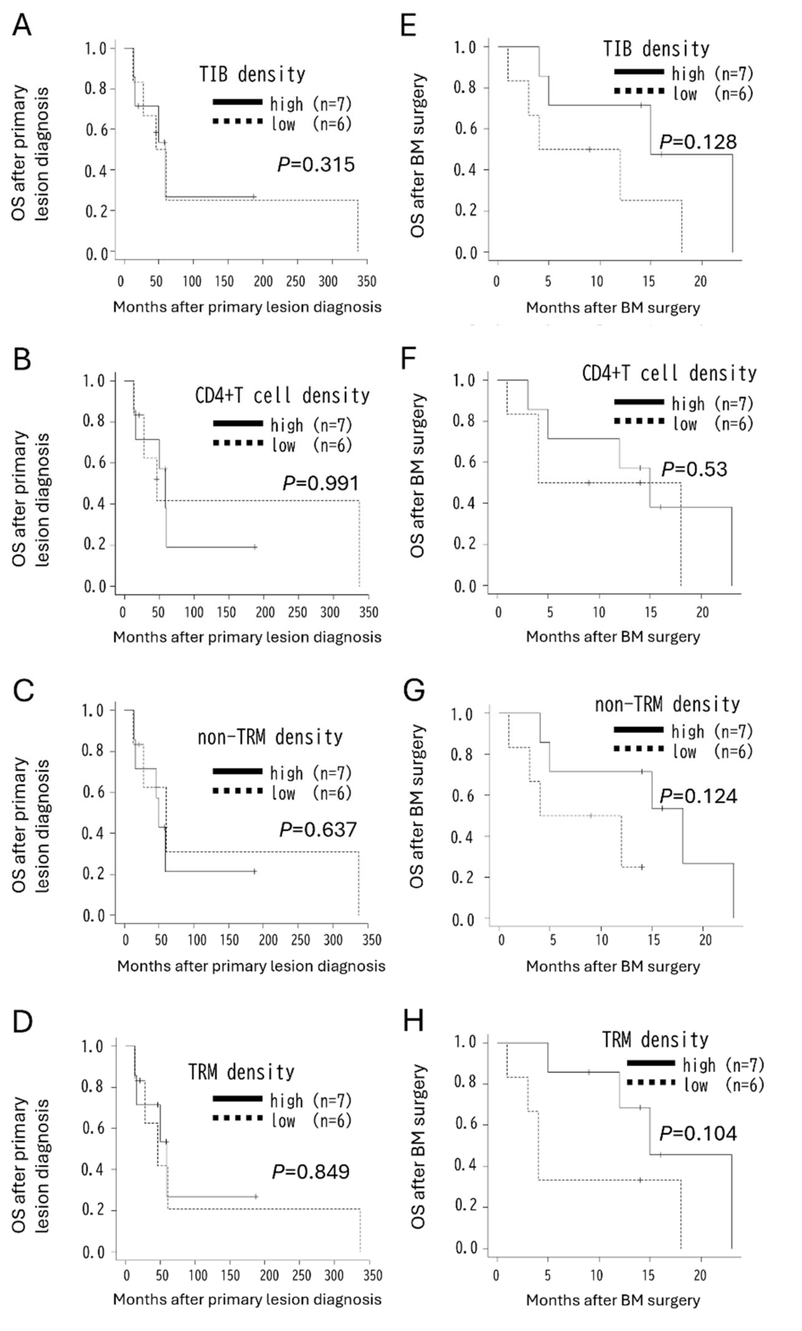

Figure S4. Additional survival analyses based on densities of each TIL subsets.

Kaplan-Meier curves for patient survival after primary lesion diagnosis (A, B, C, and D) and BM surgery (E, F, G, and H) stratified by the density (high vs. low) of (A, E) TIBs, (B, F) CD4+ T cells, (C, G) non-TRMs, and (D, H) TRMs. The patients were dichotomized into high and low groups based on the median value of each TIL subset density.
